# Mutation analysis of the *SLC26A4*, *FOXI1* and *KCNJ10* genes in individuals with congenital hearing loss

**DOI:** 10.7717/peerj.384

**Published:** 2014-05-08

**Authors:** Lynn M. Pique, Marie-Luise Brennan, Colin J. Davidson, Frederick Schaefer, John Greinwald Jr, Iris Schrijver

**Affiliations:** 1 Department of Pathology, Stanford University Medical Center, Stanford, CA, USA; 2 Department of Pediatrics, Stanford University Medical Center, Stanford, CA, USA; 3 Life Technologies, South San Francisco, CA, USA; 4 Molecular Genetics, Center for Genetic Testing at Saint Francis Hospital, Tulsa, OK, USA; 5 Divisions of Human Genetics and Otolaryngology, Cincinnati Children’s Hospital Medical Center, Cincinnati, OH, USA

**Keywords:** Pendred, MLPA, DFNB4, *SLC26A4*, *FOXI1* and *KCNJ10*, Genotyping, Genetics, SNHL

## Abstract

Pendred syndrome (PDS) and DFNB4 comprise a phenotypic spectrum of sensorineural hearing loss disorders that typically result from biallelic mutations of the *SLC26A4* gene. Although PDS and DFNB4 are recessively inherited, sequencing of the coding regions and splice sites of *SLC26A4* in individuals suspected to be affected with these conditions often fails to identify two mutations. We investigated the potential contribution of large *SLC26A4* deletions and duplications to sensorineural hearing loss (SNHL) by screening 107 probands with one known *SLC26A4* mutation by Multiplex Ligation-dependent Probe Amplification (MLPA). A heterozygous deletion, spanning exons 4–6, was detected in only one individual, accounting for approximately 1% of the missing mutations in our cohort. This low frequency is consistent with previously published MLPA results. We also examined the potential involvement of digenic inheritance in PDS/DFNB4 by sequencing the coding regions of *FOXI1* and *KCNJ10*. Of the 29 probands who were sequenced, three carried nonsynonymous variants including one novel sequence change in *FOXI1* and two polymorphisms in *KCNJ10*. We performed a review of prior studies and, in conjunction with our current data, conclude that the frequency of *FOXI1* (1.4%) and *KCNJ10* (3.6%) variants in PDS/DFNB4 individuals is low. Our results, in combination with previously published reports, indicate that large *SLC26A4* deletions and duplications as well as mutations of *FOXI1* and *KCNJ10* play limited roles in the pathogenesis of SNHL and suggest that other genetic factors likely contribute to the phenotype.

## Introduction

Hearing loss is a common congenital defect. The estimated incidence of permanent hearing loss at birth, defined as a sensorineural loss of 35 dB or more in the U.S., is one in 500 newborns (Morton & Nance, 2006). The origins of hearing loss may be genetic, environmental or multifactorial, with at least 50% of prelingual hearing loss in industrialized countries attributable to genetic abnormalities (Vele & Schrijver, 2008). Approximately 70% of prelingual hearing loss is non-syndromic; the remaining 30% is accompanied by additional clinical findings and is considered syndromic (Hilgert, Smith & Van Camp, 2009). Inheritance of hearing loss can be recessive, dominant, X-linked or mitochondrial, with autosomal recessive inheritance constituting roughly 80% of non-syndromic sensorineural hearing loss (SNHL). Mutations of a single gene, *GJB2* (OMIM *121011), which encodes the connexin 26 protein, account for the majority of autosomal recessive non-syndromic SNHL (Kenneson, Van Naarden Braun & Boyle, 2002). Mutations of the *SLC26A4* gene (OMIM *605646) are the second most frequent cause of autosomal recessive non-syndromic SNHL (Hilgert, Smith & Van Camp, 2009) and produce a phenotypic spectrum of hearing loss disorders encompassing both Pendred syndrome (PDS; OMIM #274600) and DFNB4 (OMIM #600791) (Everett et al., 1997; Li et al., 1998). *SLC26A4* is composed of 21 exons and encodes the 780 amino acid transmembrane anion transporter protein pendrin (Everett et al., 1997; Everett et al., 1999; Royaux et al., 2000; Royaux et al., 2001), which plays a key role in maintaining the endocochlear potential (Everett et al., 1999; Royaux et al., 2003).

PDS and DFNB4 are typically characterized by congenital, bilateral sensorineural hearing loss which can be progressive and is usually severe to profound. There is considerable variability of symptoms. Vestibular dysfunction as well as non-pathognomonic temporal bone abnormalities, in particular enlargement of the vestibular aqueduct (EVA), can also be present in these conditions. DFNB4, also known as non-syndromic enlarged vestibular aqueduct (NS-EVA), is not associated with other clinical findings. PDS, in contrast, classically manifests additional symptoms such as the development of an incompletely penetrant euthyroid goiter, which can be present at birth but is more likely to develop in late childhood to early adulthood. PDS is also typically accompanied by Mondini dysplasia, a reduction of the number of turns of the cochlea combined with the characteristic bilateral EVA (Schrijver & Gardner, 2006). Although the Mondini malformation can be used as a criterion for diagnosis, it is thought to be clinically heterogeneous and it remains uncertain what proportion of Mondini malformations are linked to Pendred syndrome (Reardon et al., 1997). Other, less well defined, temporal bone abnormalities can (and typically are) seen in those individuals lacking Mondini dysplasia.

PDS was originally estimated to be responsible for 7.5% of hereditary hearing loss cases (Fraser, 1965) but the actual incidence has not been determined due to difficulties inherent in diagnosing PDS, the degree of phenotypic variability (i.e., isolated hearing loss versus multisystem involvement), the frequently late onset and reduced penetrance of the goiter, and the lack of pathognomonic findings (Blons et al., 2004). Nevertheless, PDS is thought to be one of the most common forms of syndromic deafness and mutations of *SLC26A4* were reported to be the second most frequent cause of autosomal recessive non-syndromic sensorineural hearing loss worldwide (Hilgert, Smith & Van Camp, 2009).

More than 260 mutations in the *SLC26A4* gene have been identified to date (http://www.hgmd.cf.ac.uk/ac/gene.php?gene=SLC26A4), including deletions spanning multiple exons (Park et al., 2003; Hu et al., 2007; Pera et al., 2008a; Anwar et al., 2009; Siem et al., 2010). Until recently, however, individuals with SNHL and possible PDS or DFNB4 were not systematically analyzed for the presence of multiexon deletions and duplications. Multiplex Ligation-dependent Probe Amplification (MLPA) analysis of 37 probands in a Scandinavian cohort of 109 patients suspected to have PDS/DFNB4 identified a homozygous *SLC26A4* deletion of exons 4–6 in one individual, indicating that intragenic deletions and duplications may contribute to the phenotype (Rendtorff et al., 2013). Mutations of the *FOXI1* and the *KCNJ10* genes have also been associated with PDS/DFNB4 and were reported to be digenically inherited with heterozygous mutations in *SLC26A4* (Yang et al., 2007; Yang et al., 2009). *FOXI1* encodes a transcription factor that binds to the promoter region of *SLC26A4* and is responsible for upstream regulation of the gene. *KCNJ10* encodes an inwardly rectifying potassium (K^+^) channel that is involved in generating and maintaining the endocochlear potential (Marcus et al., 2002). Intragenic deletions of *SLC26A4* as well as digenic mutations with either *FOXI1* or *KCNJ10* have all been implicated in PDS/DFNB4 pathogenesis but the extent of their involvement as well as their clinical relevance for SNHL remains unclear.

We investigated the contribution of intragenic *SLC26A4* copy number changes by performing MLPA analysis on DNA samples from 107 probands with congenital SNHL who had only one identified *SLC26A4* mutation. Although it has been recommended to consider *SLC26A4* mutation analysis if there is progressive hearing loss, goiter, Mondini dysplasia, or EVA (Hilgert, Smith & Van Camp, 2009), the clinical testing strategy often adheres to the following algorithm: For individuals with congenital hearing loss that is consistent with autosomal recessive inheritance and that appears non-syndromic, diagnostic testing typically starts with *GJB2* sequence analysis. If clinical testing does not identify two pathogenic mutations, then inner ear imaging studies by MRI or CT for temporal bone anomalies may be performed. However, clinical testing of *SLC26A4* without MRI or CT is also considered acceptable because mutations in this gene are thought to be a frequent cause of SNHL (Alasti, Van Camp & Smith, 1998, updated 2012). This approach to the clinical work-up is quite common in the U.S., in part because the same DNA specimen can be used for both molecular tests sequentially and in part because imaging studies in the pediatric population can be challenging. As a result, our patients were selected based on the presence of congenital SNHL compatible with autosomal recessive inheritance, absence of two hearing loss-associated *GJB2* mutations, and the presence of only one known mutation in *SLC26A4*. In these individuals, we performed MLPA of *SLC26A4* and in a subset we also sequenced the coding regions and splice sites of *FOXI1* and *KCNJ10* to evaluate alternative etiologies.

## Materials and Methods

### Study subjects

A total of 107 individuals participated in this study. Participants provided written informed consent as appropriate. All were enrolled with IRB approval from the Stanford University Administrative Panels for the Protection of Human Subjects (IRB Approval Numbers IRB-14011 and IRB-8353). Participants included individuals from Stanford University Medical Center (*n* = 60, Stanford, CA), the Center for Genetic Testing at Saint Francis Hospital (*n* = 30, Tulsa, OK) and Cincinnati Children’s Hospital Medical Center (*n* = 17, Cincinnati, OH). All participants were probands presenting with congenital SNHL who prior to enrollment, had all received *GJB2* testing and been sequenced for mutations in the exons, splice sites and promoter region of the *SLC26A4* gene (GenBank: NC_000007.13) as part of routine clinical care. Patients who did not have two pathogenic *GJB2* mutations and who had only one identified *SLC26A4* mutation were then eligible for additional genetic testing by MLPA in an effort to identify a second disease-causing mutation. *FOXI1* (GenBank: NG_012068.1) and *KCNJ10* (GenBank: NG_016411.1) were also sequenced in a subset of the Stanford patient group (*n* = 29/60) for whom sample was available.

Because imaging analysis is not routinely ordered on children with phenotypically non-syndromic SNHL prior to *SLC26A4* sequencing, imaging analysis results were available for only a portion of the subjects (*n* = 46/107; 43%) and indicated the presence of EVA in 56% (*n* = 26/46) and Mondini malformation in none of the probands. Vestibular dysfunction complaints were noted in only one of 54 participants for whom this information was reported. Thyroid manifestations were reported in none of the Stanford probands for whom this information was available (*n* = 0/37), including five individuals older than 13 years of age.

### Detection and characterization of intragenic rearrangements

Genomic DNA samples extracted from peripheral blood by standard methods were analyzed for copy number mutations by MLPA, using the SALSA MLPA kit P280 Pendred-SLC26A4 (MRC-Holland, Amsterdam, the Netherlands) according to the manufacturer’s protocol. Capillary electrophoresis of PCR products was performed using either an ABI 310 or an ABI 3500 Genetic Analyzer (Life Technologies, Grand Island, NY, USA) and the resulting data were analyzed using GeneMarker 1.51 software (SoftGenetics, LLC., State College, PA, USA).

For breakpoint analysis in the single proband with an identified *SLC26A4* deletion, PCR products were electrophoresed on an agarose gel and the 494 base pair (bp) fragment corresponding to the deletion was excised and purified using the QIAquick Gel Extraction Kit (Qiagen, Germantown, MD). The amplicon was then sequenced on an ABI 3730xl Genetic Analyzer (Life Technologies, Grand Island, NY, USA) to confirm the breakpoints of the deletion.

### *FOXI1* and *KCNJ10* sequencing

Almost half (*n* = 29/60) of the Stanford University proband samples had direct DNA sequencing of the coding exons, including intron/exon boundaries ±20 bp into the introns, of *FOXI1* and *KCNJ10*. Primers were designed to amplify and sequence exons 1 and 2 of *FOXI1* and exon 2 of *KCNJ10* (Table 1). Amplicons were purified using the QIAquick PCR Purification Kit (Qiagen, Germantown, MD) and then sequenced on an ABI 3730xl Genetic Analyzer (Life Technologies, Grand Island, NY, USA). Sequences were compared against either the *FOXI1* (GenBank: NG_012068.1) or the *KCNJ10* (GenBank: NG_016411.1) reference sequence using Mutation Surveyor 2.51 software (SoftGenetics, LLC., State College, PA, USA). Nonsynonymous sequence variants detected in the coding regions of the *FOXI1* and *KCNJ10* genes were further analyzed using the mutation interpretation tools SIFT (Sorting Intolerant From Tolerant, http://sift.jcvi.org/) and PolyPhen-2 (Polymorphism Phenotyping v2, http://genetics.bwh.harvard.edu/pph2/), which predict the effect of an amino acid substitution on protein function.

**Table 1:** Primers used for the amplification of *FOXI1* and *KCNJ10*.

Gene	Exon	Primer name	Primer sequence (5′ to 3′)	*T*_**a**_
*FOXI1*	1	FOXI1-1F	TGAGCACCTGTCAGGGGCAG	61
*FOXI1*	1	FOXI1-1R	GAACTTTCTAGAATGGGGTCTTG	61
*FOXI1*	1	FOXI1-1Rint	CCCTGTGGGTGGAAGAAGT	55
*FOXI1*	2	FOXI1-2F	GACAATAAGGAGGAACAGAAG	55
*FOXI1*	2	FOXI1-2R	GCATGGAGGACCTCTACTG	55
*KCNJ10*	2	KCNJ10-2aF	GTTAATTCCTCCCTCCCATGG	59
*KCNJ10*	2	KCNJ10-2aR	GTTCTCCCCTTCCTTGGTTTG	59
*KCNJ10*	2	KCNJ10-2bF	GAGACCATTCGTTTCAGCCAG	59
*KCNJ10*	2	KCNJ10-2bR	AAGAAGAGGGAGTGGAGGATG	59

## Results

**Figure 1:**
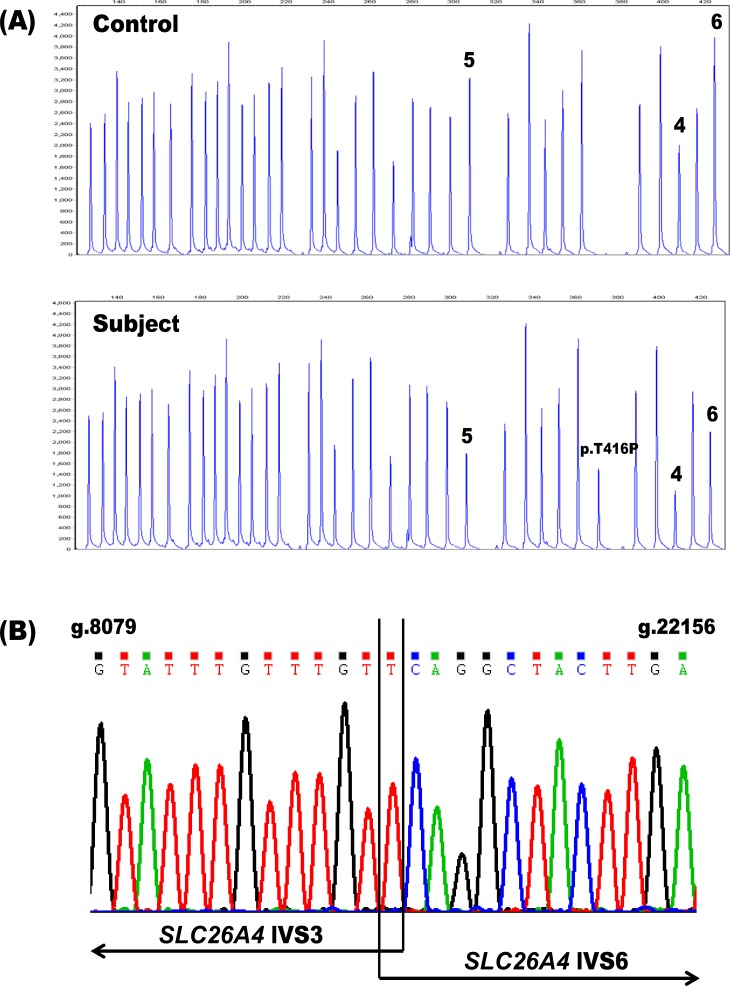
Identification of the heterozygous *SLC26A4* deletion g.8091T-22145Cdel in subject PDS41. (A) The *SLC26A4* MLPA probe mix includes probes for the 21 *SLC26A4* exons, 14 reference probes and three probes specific for the point mutations c.1001+1G>A (IV S8+1G>A), c.707T>C (p.Leu236Pro) and c.1246A>C (p.Thr416Pro). Subject PDS41 is heterozygous for a deletion (g.8091T-22145Cdel) spanning exons 4–6 in *SLC26A4*, as evidenced by the reproducible reduction in peak height for these three exon probes (peaks labeled 4, 5, 6) relative to the control. PDS41 also has a heterozygous c.1246A>C (p.Thr416Pro) mutation that was previously identified by sequencing and confirmed by these MLPA results (peak labeled p.T416P). (B) Sequencing chromatogram of the deletion breakpoints in *SLC26A4* IVS3 and IVS6. The deletion removes a total of 14,053 bp. IVS, intervening sequence.

In order to assess the potential diagnostic benefits of deletion/duplication analysis of the *SLC26A4* gene, we performed MLPA on genomic DNA samples from 107 patients with congenital hearing loss in whom only one *SLC26A4* mutation had been found by sequencing. Only one individual, PDS41, (1/107 unknown alleles; 0.93%) had MLPA results indicative of such a copy number variation—a putative heterozygous deletion spanning exons 4–6 of the gene (Fig. 1). Because deletions of these same *SLC26A4* exons were previously described in a Spanish patient (Pera et al., 2008a) and in two Norwegian patients of Lebanese descent (Siem et al., 2010; Rendtorff et al., 2013), we investigated the possibility that our MLPA assay had detected a known rearrangement in our proband. Flanking PCR primers were designed to analyze the breakpoints of the deletion (PDS IVS3 Forward: 5′-ACAATGTCCATGCCACAACC-3′ and PDS IVS6 Reverse: 5′-ACAGAGACCATTACATACATAC-3′). A duplex reaction including both the breakpoint primers and a set of control primers for the amplification of exon 4 (PDS Ex4 Forward: 5′-AGGCAAAGTCATAAGTGGAAC-3′ and PDS Ex4 Reverse: 5′-ACCTAATAGAGGTATAATGCAC-3′) resulted in two products: a 494 bp fragment corresponding to the deletion and a 289 bp fragment amplified from the control primers (data not shown). The presence of the 289 bp amplicon indicated that the deletion was indeed heterozygous. Subsequent sequencing of the 494 bp amplicon confirmed the presence of g.8091T-22145Cdel (Fig. 1). This deletion removes 14,053 bp from the *SLC26A4* gene, disrupting the open reading frame and truncating the protein at amino acid residue 105 in the first transmembrane domain.

In addition to performing MLPA analysis on samples from our patient cohort, we investigated other genes potentially contributing to their SNHL by sequencing 29 probands for variants in the coding regions of the *FOXI1* and *KCNJ10* genes. We identified a heterozygous variant of *FOXI1*, c.677C>T (p.Thr226Ile), in one patient, PDS29 (1/29 unknown alleles; 3.4%). This *FOXI1* substitution has not been previously described in SNHL. It represents a rare variant (dbSNP:rs115399307; minor allele frequency of 0.3%, http://www.ncbi.nlm.nih.gov/projects/SNP/snp_ref.cgi?rs=115399307) of unknown clinical significance. The SIFT and PolyPhen-2 programs predicted the substitution to be “tolerated” and “benign”, respectively. Two heterozygous variants of *KCNJ10* were also detected in this group (2/29 unknown alleles; 6.9%), each in a separate proband. The substitution c.812G> A(p.Arg271His) in PDS21 was predicted by SIFT and PolyPhen-2 to be “tolerated” and “benign”, respectively. This substitution is also a rare sequence variant (dbSNP: rs3795339; minor allele frequency of 0.6%, http://www.ncbi.nlm.nih.gov/projects/SNP/snp_ref.cgi?rs=3795339) of uncertain clinical significance that has been reported previously in a single heterozygous Chinese proband with non-syndromic EVA and zero mutations of *SLC26A4* (Chai et al., 2013). In that same study, however, the c.812G>A (p.Arg271His) variant was found in 10/200 or 5% of normal hearing controls, suggesting that this substitution may be a polymorphism in the Chinese population. The other *KCNJ10* variant, c.811C>T (p.Arg271Cys), was discovered in patient PDS23 and it also affects amino acid residue 271. Although this substitution is predicted to “affect protein function” by SIFT and to be “possibly damaging” by PolyPhen-2, it is a reported polymorphism of unknown clinical significance (dbSNP: rs1130183) with a minor allele frequency of 2.3% (dbSNP: http://www.ncbi.nlm.nih.gov/SNP/snp_ref.cgi?type= rs&rs=1130183). This substitution has been previously reported in three Italian individuals affected with SNHL, who also had bilateral inner ear malformations (Cirello et al., 2012) and has been associated with seizure susceptibility (Buono et al., 2004; Lenzen et al., 2005).

## Discussion

The autosomal recessive inheritance of PDS and DFNB4 is well established and yet, for many affected individuals, analysis of the coding sequences and splice sites of the *SLC26A4* gene has failed to identify one or both of the mutations required to cause these disorders (Campbell et al., 2001; Tsukamoto et al., 2003; Pryor et al., 2005; Albert et al., 2006; Yang et al., 2007; Wu et al., 2010). In fact, an analysis of six studies with a total enrollment of 769 hearing impaired probands with EVA, a non-pathognomonic clinical finding that is a hallmark of PDS and DFNB4, reveals that only 25% have biallelic *SLC26A4* mutations (Table 2). Of these same probands, 45% have at least one *SLC26A4* mutation, a percentage slightly lower than the 50% reported by GeneReviews as the proportion of PDS/DFNB4 accounted for by mutations in *SLC26A4* (http://www.ncbi.nlm.nih.gov/books/NBK1467/). Among the six included studies, there are considerable differences in the reported percentages of individuals segregating monoallelic or biallelic *SLC26A4* mutations. The proportion of probands for whom at least one *SLC26A4* mutation is detected ranges from as low as 30–40% (Campbell et al., 2001; Albert et al., 2006; Yang et al., 2007) to as high as 80–90% (Tsukamoto et al., 2003; Wu et al., 2010). The percentage of probands in whom biallelic mutations are found varies accordingly, from a low of 13% (Yang et al., 2007) to a high of 62% (Wu et al., 2010). This variation may be attributed to differences in the selection criteria of each study and/or to the patient population being tested; however, in all cases, there is a substantial proportion of individuals for whom the genotype is either incomplete or for whom no mutations in *SLC26A4* were identified at all. This has implications for genetic counseling regarding recurrence risk, whether to pursue imaging, and, more broadly, understanding of disease pathogenesis. The inability to identify both mutations in individuals suspected of having a PDS/DFNB4 hearing loss etiology suggests the possible involvement of (1) mutations in unexamined regions of *SLC26A4*; (2) mutations in other, as yet to be implicated genes; or (3) other factors, such as those that may regulate gene expression.

**Table 2:** Percentages of PDS/DFNB4 probands with *SLC26A4* mutations.

Reference	Probands	2 Mutations	1 Mutation	0 Mutations	Study selection criteria
Campbell et al., 2001	58	9 (16%)	14 (24%)	35 (60%)	Recessive HL with DVA or Mondini dysplasia
Tsukamoto et al., 2003	42	24 (57%)	10 (24%)	8 (19%)	Pendred (goiter) or bilateral HL with EVA
Pryor et al., 2005	39	14 (36%)	14 (36%)	11 (28%)	EVA in at least one ear
Albert et al., 2006	100	24 (24%)	16 (16%)	60 (60%)	Bilateral, recessive HL; EVA; no *GJB2* mutations
Yang et al., 2007	429	57 (13%)	75 (17%)	297 (69%)	HL with EVA
Wu et al., 2010	101	63 (62%)	24 (24%)	14 (14%)	Bilateral EVA
**Total**	769	191 (25%)	153 (20%)	425 (55%)	

**Notes.**

HL hearing loss DVA dilated vestibular aqueduct EVA enlarged vestibular aqueduct

The selection of study subjects is an important factor that is not consistent between studies. Our patient selection was based primarily on the genetic testing of *GJB2* and *SLC26A4* as is common in the clinical work-up for children with SNHL in the U.S., with imaging studies performed on only a subset of patients. Even in this relatively general SNHL patient population, however, we saw a considerable enrichment for probands with single *SLC26A4* mutations, compared to unaffected individuals. In the experience of the Stanford Molecular Pathology Laboratory, the frequency of heterozygous *SLC26A4* mutations in probands tested with a *GJB2* and *SLC26A4* algorithm and in whom two pathogenic mutations are not identified is 12.3%, which is significantly higher (*p* < 0.001) than the expected carrier frequency. Using a frequency of 1/500 for congenital bilateral hearing loss of ≥40 dB (Hilgert, Smith & Van Camp, 2009), and the estimate that *SLC26A4* related SNHL would account for up to 7.5%, then the frequency of such hearing loss could approximate one in 7,000. Assuming Hardy–Weinberg equilibrium, the carrier frequency would be about 1:50 (2%). This is congruent with a study in which pathogenic or possibly pathogenic mutations were identified in 1.9% of normal-hearing controls (8/428 controls; p.E29Q, p.F354S, p.F667C, p.D724G, p.G740S) (Pera et al., 2008b). An “excess” of heterozygous mutations in individuals with SNHL compared to controls has also been observed for *GJB2* (Putcha et al., 2007), a gene for which neighboring deletions can affect gene expression (Rodriguez-Paris & Schrijver, 2009).

Given that *SLC26A4* related SNHL is autosomal recessive, we postulated that these ‘missing’ *SLC26A4* mutations may be the result of intragenic deletions or duplications of one or more exons for which patients are not routinely tested. We selected 107 individuals with SNHL and monoallelic mutations of *SLC26A4* for MLPA analysis of the *SLC26A4* gene to explore this further. A handful of multiexon *SLC26A4* deletions have been described in the literature (Park et al., 2003; Hu et al., 2007; Pera et al., 2008a; Anwar et al., 2009; Siem et al., 2010) but it is unclear how many of the probands in these cohorts were tested for intragenic deletions and duplications. Recently, however, in a study of 109 Scandinavian probands with suspected PDS/DFNB4, 37 individuals with only one or zero mutations of *SLC26A4* were systematically screened for copy number variants by MLPA (Rendtorff et al., 2013). Only one harbored a homozygous deletion, of exons 4–6 (*n* = 2/63 unknown alleles; 3.2%). In our study of hearing impaired individuals with one previously identified *SLC26A4* mutation, we found an intragenic deletion in a single individual only (*n* = 1/107 unknown alleles; 0.93%). This deletion also spanned exons 4–6 of the *SLC26A4* gene and was previously detected in a patient of Spanish descent (Pera et al., 2008a). An analysis of the deletion breakpoints confirmed that our subject carried a heterozygous copy of g.8091T-22145Cdel (Fig. 1). To date, deletions and duplications seem to represent approximately 1.8% of missing *SLC26A4* mutations overall (*n* = 3/170 unknown alleles) (Rendtorff et al., 2013; this study). Despite accounting for a low percentage of the ‘missing’ mutations, clinical testing for multiexon deletions and duplications of additional patients with potential PDS/DFNB4 etiology may remain warranted in order to more firmly establish frequencies and elucidate their relative contribution to the phenotype.

Alternatively, unrecognized mutations in unexamined, noncoding regions of the gene may be responsible for the ‘missing’ *SLC26A4* mutations and contribute to the PDS/DFNB4 phenotype. For example, intronic mutations may create cryptic splice sites and mutations in the promoter region may disrupt the binding of regulatory elements. Indeed, a *cis*-regulatory element that binds transcription factor FOXI1 has been described in the *SLC26A4* promoter region (Yang et al., 2007). The regulatory element consists of two head-to-head binding sites, FBS1 and FBS2, and a mutation within this *cis*-element, c.-103T>C, has been shown to disrupt transcriptional activation of the gene by FOXI1. However, none of the probands included in this study carried mutations in the promoter region.

Mutations of the *FOXI1* gene itself have also been implicated in PDS and DFNB4. Monoallelic variants of *FOXI1* were documented in six patients with either PDS or non-syndromic EVA and were shown to compromise the ability of FOXI1 to transcriptionally activate *SLC26A4* (Yang et al., 2007). One of these six probands segregated the DFNB4 phenotype with one heterozygous mutation each of *FOXI1* and *SLC26A4*. This finding was consistent with the EVA phenotype observed in the *Slc26a4*^+/−^; *Foxi*1^+/−^ double-heterozygous mouse model and suggests that the transcriptional regulatory machinery of *SLC26A4* plays a role in PDS/DFNB4 pathogenesis. Mutations of *KCNJ10*, a gene that encodes a K^+^ channel protein, have also been associated with PDS/DFNB4 (Marcus et al., 2002). Protein expression studies in *SLC26A4* knockout mice have indicated that the absence of pendrin expression reduces KCNJ10 protein levels, supporting the hypothesis that deafness in the mouse model is secondary to loss of KCNJ10 function (Wangemann et al., 2004). A similar reduction of *KCNJ10* expression was observed in the stria vascularis of the inner ear in the haploinsufficient *Slc26a4*^+/−^ mouse mutant (Yang et al., 2009). In that same study, two individuals with the PDS/DFNB4 phenotype were reported to be double heterozygous for mutations of the *SLC26A4* and *KCNJ10* genes, further supporting a digenic model of inheritance.

We examined to what extent digenic inheritance may contribute to the PDS/DFNB4 phenotype by sequencing the coding regions and splice sites of *FOXI1* and *KCNJ10* in about half (*n* = 29/60) of the Stanford University probands enrolled in the study for whom enough sample was available for the additional analysis. Our initial sequencing of *FOXI1* and *KCNJ10* in these subjects resulted in three nonsynonymous variants overall; all three of these heterozygous substitutions are listed in the dbSNP database as having unknown clinical significance. Nonetheless, functional studies must be conducted to investigate the effects of the *FOXI1* variant on *SLC26A4* transcriptional activation and the impact of the two *KCNJ10* variants on K^+^ channel conductance before a determination of the pathogenicity of these three variants and their effect on SNHL phenotypes can be more definitively made.

Although unlikely, if the *FOXI1* variant and the two *KCNJ10* variants detected in our probands are indeed digenic mutations acting in conjunction with mutations of *SLC26A4*, then 3.4% and 6.9% of the missing mutant alleles in our SNHL patients would be attributable to variants in *FOXI1* and *KCNJ10*, respectively. However, the number of patients tested for *FOXI1* and *KCNJ10* variants (*n* = 29/107 total probands) is too small a sample size to merit this conclusion. Consequently, a meta-analysis of published studies in which *FOXI1* and *KCNJ10* were sequenced in SNHL patients with inner ear malformations was conducted. The meta-analysis does not support the frequencies observed in our patients (Table 3) and shows instead that, overall, 1.3% and 3.1% of suspected PDS/DFNB4 patients have variants in *FOXI1* and *KCNJ10*, respectively. In fact, considering that the *KCNJ10* variant c.812G>A (p.Arg271His) may be a polymorphism in the Chinese population and the *KCNJ10* variant c.811C>T (p.Arg271Cys) is a reported polymorphism in the dbSNP database, the frequency of *KCNJ10* variants in PDS/DFNB4 patients may be inflated by the inclusion of the Chinese and Italian probands carrying these substitutions and may actually be lower. The great majority of reported *FOXI1* and *KCNJ10* variants are from the initial studies that implicated the genes in the digenic inheritance of PDS/DFNB4 (Yang et al., 2007; Yang et al., 2009). However, the actual contribution of *FOXI1* and *KCNJ10* mutations to SNHL may be more limited, as illustrated by several subsequent studies in which either no *FOXI1* variants (Wu et al., 2010; Mercer, Mutton & Dahl, 2011; Lai et al., 2012; Chen et al., 2012; Chai et al., 2013), or no *KCNJ10* variants (Mercer, Mutton & Dahl, 2011; Chen et al., 2012) were identified.

**Table 3:** Meta-analysis of the frequency of *FOXI1* and *KCNJ10* variants in PDS/DFNB4 probands.

Gene	Population origin	Reference	Controls^1^	Inclusion criteria for *SLC26A4* sequencing	Selection criteria for *FOXI1* sequencing	Subjects with nonsynonymous variants
*FOXI1*	US/Sweden	Yang et al., 2007	250	EVA with or without Mondini dysplasia	0 or 1 *SLC26A4* mutations	6/372 (1.6%)^2^
*FOXI1*	Taiwan	Wu et al., 2010	100	Bilateral EVA with or without other IEMs	0 or 1 *SLC26A4* mutations	0/38 (0.0%)
*FOXI1*	Australia	Mercer, Mutton & Dahl, 2011	96	EVA	Phenotype only	0/44 (0.0%)
*FOXI1*	Italy	Cirello et al., 2012	80	Bilateral IEMs/family history of PS/goiter	0 or 1 *SLC26A4* mutations	1/14 (7.1%)^3^
*FOXI1*	China	Lai et al., 2012	100	EVA	0 or 1 *SLC26A4* mutations	0/8 (0.0%)
*FOXI1*	China	Chen et al., 2012		IEM’s	0 or 1 *SLC26A4* mutations	0/15 (0.0%)
*FOXI1*	China	Chai et al., 2013	200	Nonsyndromic EVA	0 or 1 *SLC26A4* mutations	0/33 (0.0%)
						**Reported *FOXI1* frequency**= **7/524 (1.3%)**
*FOXI1*	US	This study		Sensorineural hearing loss	1 *SLC26A4* mutation	1/29 (3.4%)
						**Total combined *FOXI1* frequency**= **8/553 (1.4%)**

Gene	Population origin	Reference	Controls^1^	Inclusion criteria for *SLC26A4* sequencing	Selection criteria for *KCNJ10* sequencing	Subjects with nonsynonymous variants
*KCNJ10*	China/US/ Canada	Yang et al., 2009	400	EVA with or without Mondini dysplasia	1 *SLC26A4* mutation	2/89 (2.2%)^4^
*KCNJ10*	Australia	Mercer, Mutton & Dahl, 2011	96	EVA	Phenotype only	0/44 (0.0%)
*KCNJ10*	Italy	Cirello et al., 2012	80	Bilateral IEMs/family history of PS/goiter	0 or 1 *SLC26A4* mutations	3/14 (21.4%)^5^
*KCNJ10*	China	Chen et al., 2012		IEM’s	0 or 1 *SLC26A4* mutations	0/15 (0.0%)
*KCNJ10*	China	Chai et al., 2013	200	Nonsyndromic EVA	0 or 1 *SLC26A4* mutations	1/33 (3.0%)^6^
						**Reported *KCNJ10* frequency** = **6/195 (3.1%)**
*KCNJ10*	US	This study		Sensorineural hearing loss	1 *SLC26A4* mutation	2/29 (6.9%)
						**Total combined *KCNJ10* frequency**= **8/224 (3.6%)**

**Notes.**

1 All controls had normal hearing and, unless otherwise noted, none harbored any of the novel variants.

2 Five variants (p.G258E, p.N161del, p.G258R, p.R267Q and p.G335V) were detected in six probands and were shown to compromise *SLC26A4* transcriptional activation.

3 Functional analysis of the identified novel variant (p.P239L) showed no impairment of *SLC26A4* transcriptional activation.

4 Two identified missense mutations (p.P194H and p.R348C) were shown to impair K^+^ channel conductance.

5 One previously reported variant (p.R271C) was identified in 3 probands.

6 One heterozygous variant (p.R271H) was detected in a single proband; this variant was also found in 10/200 normal hearing controls.

EVA Enlarged vestibular aqueduct IEM Inner ear malformation PS Pendred syndrome

The genetic basis of hearing loss is diagnostically challenging with over 100 genes implicated (http://hereditaryhearingloss.org). The phenotypic variability observed within the PDS/DFNB4 spectrum also complicates diagnosis with changes in the same gene, *SLC26A4*, responsible for syndromic as well as non-syndromic hearing loss. Most clinical centers have historically utilized tiered testing in the assessment of hearing loss genetic etiology. However, with the advent of large-scale massively parallel sequencing (MPS), future approaches will likely employ testing platforms that are more comprehensive, cost effective and efficient (Shearer & Smith, 2012). Additional sources of genetic mutation, such as deletions and duplications, will need to be included in these new testing approaches.
